# Tau here, tau there, tau almost everywhere: Clarifying the distribution of tau in the adult CNS

**DOI:** 10.1002/cm.21820

**Published:** 2023-12-16

**Authors:** Nicholas M. Kanaan

**Affiliations:** 1 Department of Translational Neuroscience, College of Human Medicine, Michigan State University, Grand Rapids, Michigan, USA; 2 Neuroscience Program, Michigan State University, East Lansing, Michigan, USA

**Keywords:** astrocyte, axon, microtubule-associated protein, neuron, oligodendrocyte, tau

## Abstract

The microtubule-associated protein tau has gained significant attention over the last several decades primarily due to its apparent role in the pathogenesis of several diseases, most notably Alzheimer’s disease. While the field has focused largely on tau’s potential contributions to disease mechanisms, comparably less work has focused on normal tau physiology. Moreover, as the field has grown, some misconceptions and dogmas regarding normal tau physiology have become engrained in the traditional narrative. Here, one of the most common misconceptions regarding tau, namely its normal cellular/subcellular distribution in the CNS, is discussed. The literature describing the presence of tau in neuronal somata, dendrites, axons and synapses, as well as in glial cells is described. The origins for the erroneous description of tau as an “axon-specific,” “axon-enriched” and/or “neuron-specific” protein are discussed as well. The goal of this work is to help address these specific dogmatic misconceptions and provide a concise description of tau’s normal cellular/subcellular localization in the adult CNS. This information can help refine our collective understanding of- and hypotheses about tau biology and pathobiology.

## 1 | INTRODUCTION

Tau is traditionally recognized as a microtubule-binding protein and has gained significant notoriety because it comprises hallmark pathological lesions in a group of diseases known as tauopathies, including Alzheimer’s disease (AD). Tau was discovered in the mid-1970s, when it was co-purified with microtubules from brain tissue lysates (Cleveland et al., 1977a, 1977b; Weingarten et al., 1975; Witman et al., 1976). This work, and subsequent experiments assessing the effects of tau on microtubules *in vitro*, led to assignment of tau primarily serving as a microtubule stabilizing protein. However, several studies by multiple research groups highlight a much broader functional repertoire of tau in normal physiology and disease pathogenesis, and in turn call into question whether tau solely functions to stabilize microtubules (Combs et al., 2019; Mueller et al., 2021; Qiang et al., 2018; Wang & Mandelkow, 2016). Interestingly, tau and MAP2 (another closely related microtubule-associated protein) likely evolved from duplication of a common ancestorial gene (MAP4) that included the addition of functional N-terminal domains, which may contribute to the diverse functional repertoire of tau (Fischer, 2022; Sündermann et al., 2016; Trushina et al., 2019).

**FIGURE 1:**
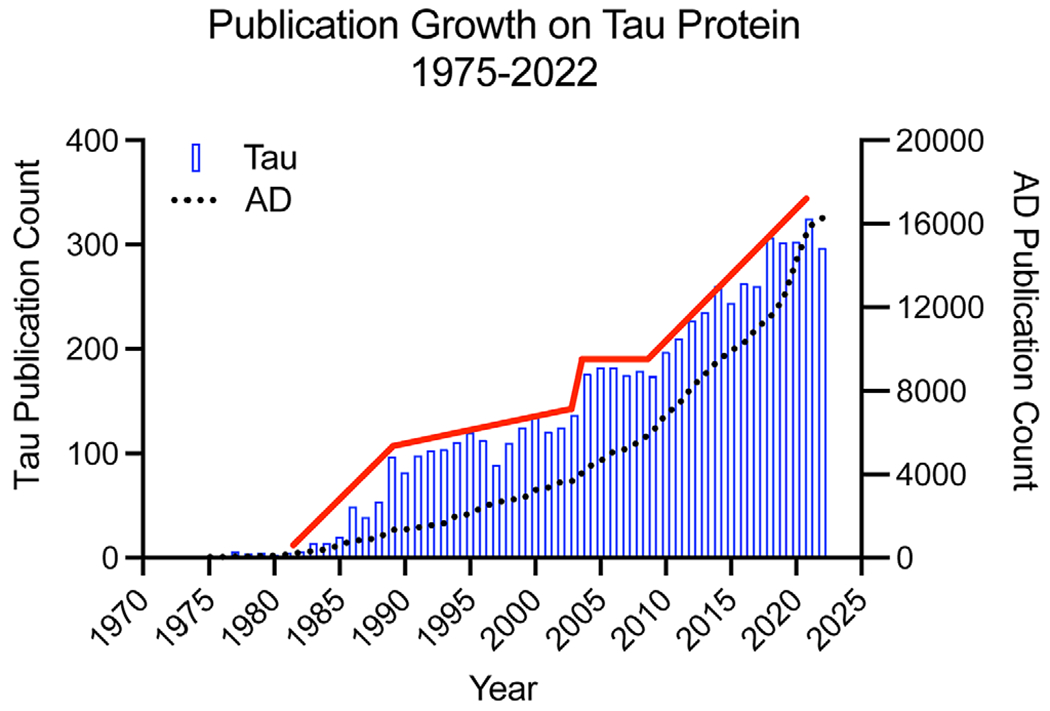
Tau protein publication growth from 1975 to 2022. Using “tau protein microtubule” as search terms in PubMed produced a total of 6,016 publications from 1975 to 2022. Several notable epochs can be identified from the yearly number of tau publications. First, around the mid to late 1980s there was a rapid increase in tau related publications, likely driven in part by the identification of tau as the main constituent of neurofibrillary tangles in Alzheimer’s disease. Second, relatively little growth was seen through the 1990s, likely due to the heavy focus in the AD field on amyloid-β and amyloid precursor protein. Finally, starting in approximately the early 2000s (shortly after the discovery of mutations in the *MAPT* gene that cause inherited tauopathy) and through the present day there was a relatively rapid expansion of the tau field. The red line was added to show the general trend in growth of tau-specific publications. In contrast, using the search term “Alzheimer’s disease” (black dashed line) shows a smoother and continual exponential growth in the number of publications through this time.

Since its discovery, research on tau has waxed and waned but overall, it has continued to grow over the years (Figure 1). Specifically, shortly after the discovery of tau there was a period of growth (particularly in the early to mid-1980s) due, at least in part, to the development of highly specific monoclonal antibodies that facilitated detailed investigation of physiological and pathological tau (more discussion below). This era of productive research on tau was followed by an ebb in the 1990s, likely driven, at least in part, by a strong focus on amyloid precursor protein and amyloid-β in the AD field. As interests beyond amyloid began to gain a foothold and the discovery that tau mutations cause inherited neurodegenerative diseases emerged (Hutton et al., 1998), the tau field started to expand in the early 2000s and the field has enjoyed relatively rapid growth since. In comparison, the growth of publications generally related to AD shows a smoother exponential growth over the same period. While the increase in tau-focused publications brought much needed attention to this important protein and its role in normal physiology and disease pathobiology, it also came with growing pains in the form of erroneous dogmas and misconceptions. Specifically, the notion that tau is a neuron- and axon-specific protein remains a pervasive idea in the field, despite robust evidence to the contrary dating back to some of the earliest studies on tau protein localization (~10 papers from 1987 to 2023 accurately highlight aspects of tau distribution) (Brady et al., 1995; Bullmann et al., 2009; Gartner et al., 1998; Kanaan & Grabinski, 2021; LoPresti et al., 1995; Migheli et al., 1988; Papasozomenos & Binder, 1987; Paterno et al., 2022; Takahashi et al., 2000; Tashiro et al., 1997). In an attempt to clarify these misconceptions, the following discussion provides an explicit description of the normal distribution of tau in the adult CNS and the contributing factors to the misconceptions on tau localization.

## 2 | A BRIEF HISTORY ON TAU DISTRIBUTION

A large body of literature exists regarding the pathological accumulation of tau in human diseases; however, much less work exists that describes the normal distribution of tau in the CNS. Tau localization in the CNS of naïve animals was first investigated about a decade after its discovery. At the center of this work was the development of the first monoclonal tau-specific antibody, aptly named Tau1, by Dr. Lester “Skip” Binder (Binder et al., 1985). Using the Tau1 antibody, the Binder group studied tau distribution in rat and bovine brains. These initial studies reported that tau is widely distributed in the CNS, but they found no evidence of normal tau localization in neuronal somata and dendrites or in non-neuronal cells (i.e., glial cells) (Binder et al., 1985; Binder et al., 1986; Busciglio et al., 1987; Dotti et al., 1987). Indeed, this collective work is the first appearance in the literature of the idea that tau is an axon- and neuron-specific protein in the adult CNS.

Around this time, the field was starting to uncover that tau was likely the main protein constituent of neurofibrillary tangles (NFTs), one of the hallmark pathological lesions in AD. Initial immunohistological stains using polyclonal rabbit anti-tau antibodies (raised against insoluble paired-helical filaments of tau) showed reactivity with NFTs, neuropil threads and neuritic plaques (Brion et al., 1985; Ihara et al., 1986; Kosik et al., 1986; Nukina & Ihara, 1986). Shortly after these publications, and armed with the first anti-tau monoclonal antibody, the Binder group and collaborators tested Tau1 reactivity in AD brain sections (Grundke-Iqbal et al., 1986; Wood et al., 1986). Surprisingly, Tau1 immunoreactivity in AD tissue showed restricted labeling of the pathological lesions (e.g., NFTs and neuritic plaques) in comparison to the polyclonal antibodies published earlier. The disconnect in staining patterns between Tau1 and the polyclonal tau antibodies led the investigators to test whether phosphorylation of the tau protein (tau was recognized as a potential phosphoprotein since its discovery; Cleveland et al., 1977a), may be masking the Tau1 epitope. The Binder group treated tissue sections with a phosphatase to dephosphorylate tau and then repeated the Tau1 stains. The results were striking. The level of Tau1 reactivity was significantly improved and better matched the prior results obtained with the polyclonal tau antibodies. These studies ultimately led to the discovery that Tau1 antibody reactivity was negatively impacted by phosphorylation at its epitope. This seemingly simple discovery would ultimately lead to a revisitation of the pattern of Tau1 immunoreactivity and the first accurate description of the physiological distribution of tau in the CNS (Papasozomenos & Binder, 1987).

With the new understanding that Tau1 reactivity was blocked by phosphorylation, the Binder group re-evaluated the physiological distribution of tau in the adult CNS using immunohistological approaches. The results were extremely clear, the proper use of Tau1 (i.e., in phosphatase-treated sections) highlighted tau throughout neuronal somata, dendrites and axons, as well the nuclei of neurons. Moreover, tau was noted in glial cells as well, which they concluded were likely astrocytes (based on location and morphology) and perineuronal glia (Papasozomenos & Binder, 1987). Subsequent studies by multiple groups spanning the subsequent 35+ years, have used various tau antibodies to further explore the physiological distribution of tau in the adult CNS and confirm the widespread distribution of tau throughout neuronal compartments and in glial cells (primarily interfascicular and perineuronal oligodendrocytes) (Brady et al., 1995; Bullmann et al., 2009; Gartner et al., 1998; Irving et al., 1996; Kanaan & Grabinski, 2021; Kubo, Misonou, et al., 2019; Loomis et al., 1990; LoPresti et al., 1995; Migheli et al., 1988; Paterno et al., 2022; Takahashi et al., 2000; Tashiro et al., 1997; Thurston et al., 1996; Thurston et al., 1997).

**FIGURE 2:**
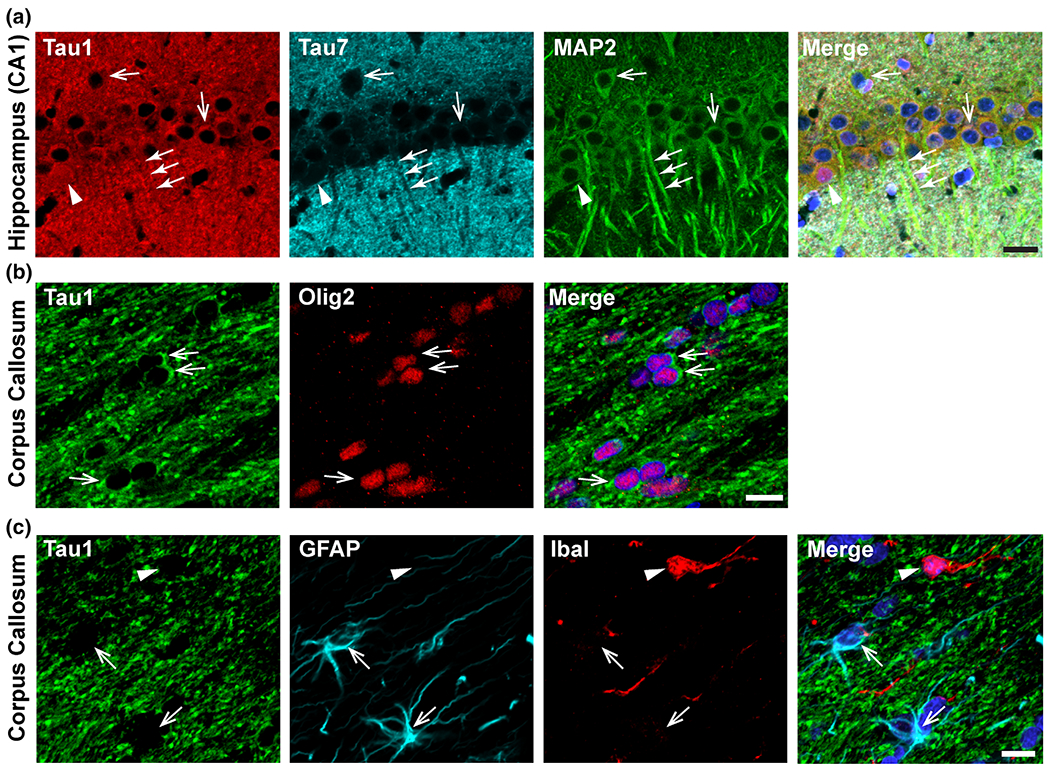
Cellular distribution of tau in the rat CNS. Representative multi-label immunofluorescence images from the rat brain (14 months old, Fisher 344 rats) illustrate the localization of tau under physiological conditions. The hippocampus (a) and corpus callosum (b and c) in tissue sections treated with phosphatase to remove phosphorylation at the Tau1 antibody epitope are shown. (a) In the hippocampus, Tau1 (red; epitope: dephosphorylated aa 192–204) demonstrates similar levels of tau protein in the somata (open arrows) and dendrites (closed arrows) of neurons and in the nucleus of a subset of neurons (arrowhead). Note the staining intensity in the somata is similar to the parenchymal staining (dendrites and axons). In contrast, the Tau7 antibody (cyan; epitope: aa 430–441) labels parenchymal tau, but does not label somatodendritic tau. MAP2 (green) was used to highlight the somatodendritic compartment of neurons. (b) Tau1 antibody labels mature oligodendrocytes (green, open arrows, Olig2 antibody). (c) Tau1 antibody does not readily label astrocytes (cyan, open arrows, GFAP antibody) or microglia (red, arrowhead, IbaI antibody). Merged images include DAPI nuclear counterstains. Scale bars are 20 μm in (a) and 10 μm in (b, c). Adapted from Kanaan and Grabinski (2021).

We recently confirmed the physiological distribution of tau in the adult rat (Figure 2) and monkey brain using a panel of tau antibodies with different epitopes (Kanaan & Grabinski, 2021). We used Tau1 (unphosphorylated aa 192–204), Tau5 (aa 210–230, unaffected by phosphorylation), Tau7 (aa 430–441, unaffected by phosphorylation), polyclonal R1 (mixed epitopes, unaffected by phosphorylation), NT9 (aa 9–45, unaffected by phosphorylation) and NT15 (aa 103–155, unaffected by phosphorylation). In adult rat and monkey brains, physiological tau is localized in the neuronal somatodendritic and axonal compartments (Figure 2). Tau is also present in neuronal nuclei, but interestingly, this is only observed in a subset of neurons (Figure 2), perhaps indicating that nuclear localization of tau is transient, context-dependent and/or cell-type dependent. Deciphering the factors dictating the occurrence and the role of nuclear tau is actively being investigated, but nuclear tau may participate in chromatin stabilization, nucleolar organization and/or protecting DNA and RNA from damage (Camero et al., 2014; Sjöberg et al., 2006; Sultan et al., 2011; Violet et al., 2014; Wei et al., 2008). Comparing tau levels in somatodendritic versus axon enriched regions of the hippocampus revealed that, depending on the specific antibody used, levels of somatodendritic tau are relatively equal to, higher than, or lower than axonal tau (Kanaan & Grabinski, 2021). These results highlight differential labeling of tau with different antibodies and stress the importance of using multiple assays to ask the same question. This is likely another contributing factor to the dogmatic misconception of tau’s localization (see below). If one were to characterize the cellular distribution of tau with an antibody that only labels a subset of all tau proteins, they may come to a different conclusion than results obtained with a different antibody. Finally, although a few studies have failed to find synaptic tau, several others report tau in dendritic spines and synapses, which may play a role in tau-mediated synaptic dysfunction in disease (Frandemiche et al., 2014; Ittner et al., 2010; Jadhav et al., 2015; Kubo, Misonou, et al., 2019; Mondragon-Rodriguez et al., 2012; Papasozomenos & Binder, 1987; Schaler et al., 2021; Tai et al., 2012) (reviewed in (Hanger et al., 2019; Robbins et al., 2021).

In addition to neurons, tau also is strongly expressed in mature oligodendrocytes (Figure 2) (Kanaan & Grabinski, 2021; LoPresti, 2002), where it may participate in myelination through Fyn kinase regulation, and likely other yet to be defined oligodendrocyte functions (Belkadi & LoPresti, 2008; Klein et al., 2002; Lee et al., 1998; LoPresti, 2002, 2015; Seiberlich et al., 2015). In contrast, oligodendrocyte precursor cells, astrocytes or microglia display little to no tau expression (Figure 2). There are few detailed investigations of physiological astrocyte tau expression but most studies have found it to be limited or non-existent (Kanaan & Grabinski, 2021; Kubo, Misonou, et al., 2019). High tau expression in neurons and oligodendrocytes, low expression in astrocytes and little to no expression in microglia is supported by RNAseq studies as well (Habib et al., 2016; Ximerakis et al., 2019). The inconsistencies in the literature regarding immunohistochemical assessments of astrocytic tau expression may again highlight how the selected antibody determines detection. Alternatively, there may be brain region-specific and/or context-dependent expression of tau in astrocytes. Currently, the physiological and/or pathological roles of endogenous astrocytic tau are not well defined, but tau expression may cause reactivity and dysfunction in astrocytes (Dabir et al., 2006; Ezerskiy et al., 2022; Forman et al., 2005). Finally, tau distributions are similar across rat and monkey tissues, demonstrating that tau’s cellular and subcellular localization in neurons and glia is conserved from rodents to primates (Kanaan & Grabinski, 2021).

Collectively, the existing literature demonstrates the physiological distribution of tau in the adult CNS includes the localization in neuronal somata, dendrites, synapses, and axons, as well as mature oligodendrocytes and potentially astrocytes. The full landscape of tau distribution is not revealed by all antibodies, but the fact that antibodies with different epitopes produce different patterns of immunoreactivity may indirectly shed light onto normal tau physiology. These differences suggest there is differential availability of epitopes due to post-translational modifications, conformation, and/or potentially epitope masking from protein binding partners in different neuronal compartments. This is an exciting concept that underscores the dynamic nature of tau physiology. For example, the lack of staining in the somatodendritic compartment with Tau7, an extreme C-terminal antibody, suggests its epitope is unavailable for binding in the somata and dendrites, despite the presence of abundant levels of tau protein in these compartments (Kanaan & Grabinski, 2021). The implications of such location-dependent differences in the state of tau await further investigation. An additional layer of complexity that requires further investigation is to clearly define the subcellular distribution of the six primary CNS isoforms, as well as the big tau isoform found primarily in the PNS and selective CNS regions (Boyne et al., 1995; Fischer, 2023; Fischer & Baas, 2020). Nonetheless, the widespread localization of tau certainly supports a diverse functional repertoire, well beyond regulating microtubule dynamics (reviewed in; Baas & Qiang, 2019; Combs et al., 2019; Mueller et al., 2021; Wang & Mandelkow, 2016). The existing body of work on tau distribution has set the stage for the field to reassess the physiological localization of tau in the adult CNS. A more precise understanding of where tau is normally located will enable a more nuanced interpretation of data regarding a shift in tau localization (whether between neuronal compartments or different cell types) in disease conditions.

## 3 | WHY HAVE MISCONCEPTIONS REGARDING TAU DISTRIBUTION PERSISTED?

The description of tau as being “axon-specific” or “axon enriched,” with corresponding descriptions of somatodendritic tau being “negligible,” are based on experimental observations. Many of these observations come from work in primary neuron cultures. Early studies noted tau’s ubiquitous presence throughout the somatodendritic and axonal compartments of primary neurons in culture (grown for 3–4 days in vitro) or during early neurodevelopment *in vivo* (using either Tau1 without dephosphorylation or the 7A6 polyclonal tau antibody) (Brandt et al., 1995; Dotti et al., 1987; Ferreira et al., 1987; Kempf et al., 1996). However, as neurons matured into early postnatal ages there was a clear shift in Tau1 signal towards axonal specificity (tissues were not dephosphorylated) (Ferreira et al., 1987). Now coupled with the studies showing Tau1 sensitivity to phosphorylation (see above), we know the “axon-specificity” noted in these early studies with Tau1 was due to changes in tau phosphorylation at the Tau1 epitope, not a lack of its presence in the somatodendritic compartment.

Several more recent studies report axon-specific localization of endogenous tau in cultured neurons (Bachmann et al., 2021; Balaji et al., 2018; Bell & Zempel, 2022; Li et al., 2011; Tjiang & Zempel, 2022; Zempel et al., 2010). While these studies clearly demonstrate axon-specific signal for tau, they are limited by the primary use of a single tau antibody, K9JA, which is described as a C-terminal antibody. Our work using Tau7, another C-terminal antibody, clearly demonstrates that antibodies in this region may only label axonal tau, despite its presence in the soma, dendrites and nucleus (Kanaan & Grabinski, 2021). Thus, once again the axon-specificity described in these studies is likely a result of the specific reagent used. Moreover, additional studies also describe an enriched level of axonal tau in more mature cultured neurons (7 days *in vitro*) using the Tau1 antibody (Black et al., 1996; Mandell & Banker, 1996; van Beuningen et al., 2015). However, the cells were not dephosphorylated prior to staining, eliminating the ability to effectively label somatodendritic tau with Tau1 antibody. In unpublished work, we have observed abundant somatodendritic tau using several different tau antibodies in cultured primary neurons from wild-type rats or human tau knock-in mice, including Tau1 (with dephosphorylation treatment), Tau5, R1, Tau A19560 (Abclonal), Tau D1M9X (CST), and Tau12, among others.

The other major body of work that demonstrates axonal enrichment of tau in cultured neurons utilizes exogenous expression of fluorescently labeled tau to visualize its subcellular localization (Bachmann et al., 2021; Bell & Zempel, 2022; Bell-Simons et al., 2023; Zempel et al., 2017). In these studies, fluorescently labeled tau is co-transfected with tdTomato, and tau fluorescence in distinct subcellular compartments is normalized to tdTomato in the same compartments. Expression of exogenous tau typically produces high levels throughout the soma, dendrites and axons of neurons. In contrast, tdTomato distribution is variable between different subcellular compartments, with high levels in the soma and dendrites and low levels in the axons (see above references). As levels of tdTomato are used to normalize levels of tau within subcellular compartments, the uneven distribution of tdTomato produces difficulty in effectively comparing axonal to somatodendritic tau levels. Thus, conclusions are limited to the differential distribution of tau in comparison to tdTomato, not the physiological compartmental enrichment of tau. Collectively, these studies show that reagent-specific selectivity likely underlies the observation that tau is “axon-specific” or “axon-enriched.” Nonetheless, the gold standard for determining tau distribution should be tissue from *in vivo* models (or human tissues), and accurately recapitulating the *in vivo* findings is the bar that other alternative model systems (e.g., primary neurons, iPSCs, etc.) must meet, not the other way around.

Additional support for these misconceptions is garnered by studies using antibodies that do not effectively highlight the full distribution of tau in the CNS, often not effectively labeling tau present in the soma and dendrites (as described above). The unmyelinated mossy fibers projecting from the dentate gyrus to the CA3 pyramidal neurons in the hippocampus is a visually striking example of high axonal tau (Kanaan & Grabinski, 2021; Kubo, Misonou, et al., 2019; Kubo, Ueda, et al., 2019). However, once again this differential staining pattern is not readily apparent with antibodies that highlight somatodendritic tau (e.g., Tau1, R1), where somata and axons display similar tau levels (Kanaan & Grabinski, 2021). In addition, studies have shown that some axonal populations display weak tau staining *in vivo*, further challenging the assertion that tau is axon-specific or enriched (Kubo, Misonou, et al., 2019; Kubo, Ueda, et al., 2019; Paterno et al., 2022). Finally, many papers do not cite the corrected conclusions on tau distribution published by the Binder group in the 1980s. Again, the literature supports the notion that the reagents of choice can significantly impact what is seen and subsequent conclusions drawn.

Finally, additional layers of complexity are added when one considers other technical aspects of staining that may alter epitope reactivity and protein distributions. For example, antigen retrieval (e.g., boiling, enzymatic, etc.) can modify protein conformation and/or the display of certain epitopes. Different fixatives (e.g., paraformaldehyde, glutaraldehyde, methanol, etc.) and methodological variables (e.g., paraffin embedding, fixation timing, etc.) may alter antibody reactivity. Certainly, these issues deserve further attention in the field as it continues to grow and build our understanding of tau physiology and distribution.

## 4 | IMPACT OF THESE MISCONCEPTIONS

Despite the published corrections on tau distribution by the Binder group (Papasozomenos & Binder, 1987) and subsequent tissue and culture studies noted above, the ideas that tau is neuron-specific and axon-specific form the basis for interpretations of experimental results and hypotheses regarding disease processes. For instance, a prominent hypothesis in the AD field is that tau undergoes an abnormal redistribution or mislocalization from the axonal to the somatodendritic and synaptic compartments, and this represents a critical pathological step in the pathogenesis of AD and other tauopathies. Importantly, the physiological presence of somatodendritic and synaptic tau does not directly eliminate the possibility that tau mislocalization might occur and contribute to disease pathogenesis. In fact, several studies suggest retrograde diffusion of axonal tau, which is normally prevented by the axon initial segment, may be intimately linked to tau pathobiology (Bell et al., 2021; Li et al., 2011; Sohn et al., 2016; Zempel et al., 2017; Zempel & Mandelkow, 2019), but the normal presence of substantive levels of tau in these compartments must be considered. The accumulation of pathological tau species in the somatodendritic or synaptic compartment does not prove mislocalization or redistribution. Indeed, the extent to which modifications of the tau already within the soma and dendrites or the abnormal retrograde diffusion of modified axonal tau contribute to the observed accumulation of somatodendritic tau in disease remains unresolved and may be difficult to study. Finally, the abundant levels of tau normally seen in mature oligodendrocytes require that we no longer consider tau a neuron-specific protein. Tau likely plays an important functional role in oligodendrocytes, and potentially astrocytes, under physiological and disease conditions, which requires further investigation to better understand.

## 5 | CONCLUDING REMARKS

In the realm of molecular neurobiology, we perceive what our reagents detect. As such, it is imperative that we, as a scientific community, draw from the totality of our resources and available information when interpreting data and making conclusions. The discussed studies highlight the importance of using comprehensive assessments of tau (i.e., multiple tau antibodies with different epitopes) when studying tau distribution. Skip Binder used to say, “if anyone knew anything about tau, they would never choose to study it.” While the comment was typically made in gest, the underlying concepts that tau is extremely diverse, complex and can be difficult to effectively study remains a truth. Tau has many secrets that the field will undoubtedly continue to discover, but we must move forward with an accurate understanding of where tau normally exists to better appreciate what it might be doing both in physiological and pathological contexts. Increased depth of studying the distribution of tau with improved resolution throughout individual cells and all brain regions would continue to strengthen our understanding of where this protein resides and may require flexibility in our definitions of tau distribution in the CNS. Moreover, the increased attention towards tau in a broad set of cellular contexts and functions is exciting and the field has a bright future of deepening our understanding of this special protein.

## Data Availability

The data that support the findings of this study are available from the corresponding author upon reasonable request.
